# Nesidioblastosis in Patients With Severe Postbariatric Hypoglycemia: A Monocentric Case Series

**DOI:** 10.1210/jendso/bvaf125

**Published:** 2025-07-30

**Authors:** Danielle Dawes, Premal Trivedi, Helen Lawler

**Affiliations:** Division of Endocrinology, Metabolism and Diabetes, University of Colorado School of Medicine, Aurora, CO 80045, USA; Division of Vascular and Interventional Radiology, University of Colorado School of Medicine, Aurora, CO 80045, USA; Division of Endocrinology, Metabolism and Diabetes, University of Colorado School of Medicine, Aurora, CO 80045, USA

**Keywords:** postbariatric hypoglycemia, nesidioblastosis, pancreatectomy, SACST

## Abstract

**Purpose:**

Partial pancreatectomy has been explored as a treatment for postbariatric hypoglycemia (PBH) yet is often ineffective due to recurring hypoglycemia. Thus, the role of nesidioblastosis in PBH's pathophysiology is controversial. Complicating matters, nesidioblastosis may be present but difficult to identify on histopathology. We aim to present data and review the literature to understand the role of nesidioblastosis in PBH and assess the efficacy of pancreatic surgery for PBH treatment.

**Methods:**

Between 2010 and 2024, 7 patients with severe PBH underwent selective arterial calcium stimulation tests (SACSTs) to assess for nesidioblastosis. Subsequently, 5 of these patients underwent pancreatic surgery. Histopathological findings were reviewed. Patient characteristics and outcomes were reported. A literature review was also performed.

**Results:**

All 7 patients (100%) had SACST results consistent with nesidioblastosis. Four patients pursued partial pancreatectomy, and 1 pursued total pancreatectomy. Pancreatic histopathology showed nesidioblastosis in 2 of the 5 surgical cases. Hypoglycemia initially resolved in 3 of the 4 patients who underwent partial pancreatectomy but later recurred after 1 year in 1 patient and after 7 years in the other 2. One patient had minimal improvement leading to completion pancreatectomy. This patient and the patient who opted for initial total pancreatectomy experienced hypoglycemia resolution but developed insulin-dependent diabetes.

**Conclusion:**

Nesidioblastosis may play a role in the pathophysiology of PBH, as suggested by the SACST findings and temporary hypoglycemia resolution after surgery. However, hypoglycemia recurrence suggests other pathological mechanisms primarily contribute to PBH. Overall, our results show long-term inefficacy of partial pancreatectomy as a treatment for PBH.

Postbariatric hypoglycemia (PBH) is a condition characterized by hyperinsulinemic postprandial hypoglycemia, typically emerging 1 year after bariatric surgery. Since its initial description by Service et al in 2005, PBH has been recognized as a significant postsurgical complication, affecting up to 30% of patients who undergo bariatric or upper gastrointestinal surgery with Roux-en-Y gastric bypass (RYGB) being the most associated procedure [1, 2]. The condition arises from surgical alterations in the gastrointestinal anatomy leading to complex physiological changes including alterations in gut hormones such as glucagon-like peptide 1 (GLP-1), enhanced glucose absorption, increased insulin secretion, altered bile acid metabolism, and a dysfunctional counterregulatory response to hypoglycemia [3-5].

Symptom onset occurs at least 6 months postoperatively, and hypoglycemic episodes are typically postprandial, occurring 1 to 3 hours after eating meals. Episodes are usually worsened by meals containing simple carbohydrates. Severe PBH refers to cases in which hypoglycemic episodes are resistant to standard therapies of dietary changes and pharmacotherapy with neuroglycopenic symptoms (ie, confusion, slurred speech, loss of consciousness, seizures) occurring multiple times per week, causing a significant impairment in quality of life [6].

Despite growing research on PBH, its underlying pathophysiology remains incompletely understood. The role of nesidioblastosis, defined as an increase in pancreatic islet cell mass and diffuse hyperplasia of pancreatic β cells, is a particularly controversial aspect of this condition. Nesidioblastosis has been hypothesized to develop from increased secretion of gastrointestinal hormones, such as GLP-1, stimulating β-cell growth and eventually contributing to hypoglycemia by exaggerated insulin release [4, 5, 7]. Rabiee et al conducted a hyperglycemic clamp study with GLP-1 infusion in a patient with PBH, demonstrating exaggerated insulin secretion and impaired glucagon release, both of which were attenuated by octreotide treatment and distal pancreatectomy [8]. Histologic analysis of the pancreas revealed hypertrophy and ectopic location of β cells, likely driven by overexpression of the islet cell transcription factor PDX-1, a consequence of prolonged GLP-1 hypersecretion [8]. However, the role of nesidioblastosis in PBH remains uncertain, with many studies suggesting that alternative mechanisms are more significant. Complicating matters, there are no universally accepted histopathological criteria for diagnosing nesidioblastosis in adults. Diagnosis is often based on a constellation of suggestive features, such as islet hypertrophy, irregularly shaped or enlarged islets, and ductuloinsular complexes, but these findings can be subtle, variably present, and subject to interpretation [9, 10]. As a result, pathology assessment requires integration with clinical history and often remains somewhat subjective.

A selective arterial calcium stimulation test (SACST) is a diagnostic tool used to evaluate hyperinsulinemia in patients with PBH or other forms of hyperinsulinemic hypoglycemia such as insulinoma or noninsulinoma pancreatogenous hypoglycemia syndrome [11]. During SACST, a sampling catheter is advanced into the right hepatic vein to measure insulin levels, while an injection catheter delivers calcium gluconate into different pancreatic arteries sequentially [11]. Calcium gluconate acts as an insulin secretagogue, stimulating insulin release specifically from insulinomas or areas of islet cell hyperplasia but notably not from normal β cells where the glucose and insulin levels remain unchanged with calcium gluconate infusion [12]. A significant rise in insulin levels, typically at least 2 to 3 times the baseline, indicates the presence of hyperfunctioning islets. If this increase is localized to a single artery or region, it suggests a focal abnormality such as an insulinoma. However, if the response is observed across multiple arteries, it points toward a more diffuse pathological process of nesidioblastosis [11, 13].

Historically, partial pancreatectomy was explored as a treatment for patients with severe PBH and noninsulinoma pancreatogenous hypoglycemia syndrome. However, while early studies showed some initial benefit, pancreatectomy has largely fallen out of favor as a treatment option for PBH due to high recurrence rates of hypoglycemia following surgery [14]. A literature review found pancreatectomy resolved hypoglycemia in only 54% of cases, while a survey reported that 90% of patients had recurrence of hypoglycemia [15, 16]. Interestingly, 75% still reported improved quality of life after the surgery [16].

This case series reviews the current literature on the relationship between nesidioblastosis and PBH, analyzes the prevalence of nesidioblastosis in 7 patients with severe PBH at our institution, and presents the clinical characteristics and outcomes from those patients who underwent pancreatic surgery as a treatment for this challenging condition. By analyzing calcium stimulation tests, histopathological findings, and clinical outcomes following pancreatectomy, we aim to contribute to the ongoing discussion about the potential role of nesidioblastosis in PBH and to provide long-term efficacy data on patients who have undergone pancreatic surgery for the treatment of PBH. While this case series is not intended to draw definitive conclusions or establish causality, our goal is to highlight clinical outcomes and raise questions that may guide future research and patient care.

## Methods

This case series includes 7 patients treated for severe PBH at our institution between 2010 and 2024. PBH was diagnosed in these patients with the following criteria: postprandial hypoglycemia occurring at least 6 months after bariatric surgery, documented by plasma glucose <55 mg/dL associated with symptoms. All patients also had negative imaging for insulinoma and/or negative prolonged fasting tests. SACSTs were performed in 7 patients, and 5 of these patients underwent subsequent partial or complete pancreatectomy. Data was obtained through a comprehensive retrospective chart review. Given the nature of the study, it was deemed exempt from institutional review board review in accordance with institutional policies as it involved retrospective analysis of de-identified patient data without direct patient interaction.

## Results

### Clinical Characteristics

All 7 patients with PBH were female and underwent RYGB at a mean age of 44.1 years (range: 30-57), with a mean maximum body mass index of 40.2 kg/m² (range: 30.5-48) and a mean nadir body mass index of 19.9 kg/m² (range: 17.5-24). The mean onset of PBH occurred 2.9 years (range: 0.5-2) after RYGB (Table 1). Only 1 patient (case 1) had type 2 diabetes prior to surgery, which went into remission post-RYGB. All 7 patients experienced severe PBH, characterized by failure of hypoglycemia to improve with dietary modifications and multiple pharmacotherapies, leading to persistent neuroglycopenic symptoms occurring multiple times per week and significantly impaired quality of life.

**Table 1.** Patient characteristics

Case	Age at bariatric surgery (years)	Type of surgery	Sex	% body weight lost	BMI max (kg/m²)	BMI nadir (kg/m²)	Diabetes history prior to RYGB	Cholecystectomy prior to RYGB	SSRI use	Time to PBH onset (years)
1	48	RYGB	F	58	48	20	T2DM on insulin	Yes	Yes	2.5
2	57	RYGB	F	53	39	18.5	None	Yes	No	2
3	41	RYGB	F	60	46	18.5	Pre-DM	No	Yes	3
4	43	RYGB	F	49	47	24	Pre-DM	Yes	Yes	0.5
5	44	RYGB	F	53	37	17.5	None	No	Yes	2
6	30	RYGB	F	38	30.5	19	None	Yes	Yes	2
7	46	RYGB	F	37	34	21.5	None	No	Yes	9

Abbreviations: BMI, body mass index; F, female; PBH, postbariatric hypoglycemia; pre-DM, prediabetes mellitus; RYGB, Roux-en-Y gastric bypass; SSRI, selective serotonin reuptake inhibitor; T2DM, type 2 diabetes mellitus.

Previous studies have identified several risk factors for PBH, including RYGB, female sex, absence of diabetes, use of selective serotonin reuptake inhibitor/serotonin-norepinephrine reuptake inhibitor medications, history of cholecystectomy, and self-reported hypoglycemia symptoms before surgery [2, 17-19]. Our cohort exhibited many of these risk factors: all patients were female and had undergone RYGB. A history of cholecystectomy before RYGB was present in 4 cases, and 6 patients were on selective serotonin reuptake inhibitor or serotonin-norepinephrine reuptake inhibitor therapy.

### Results of SACSTs

Seven patients underwent a total of 9 SACST procedures, as 2 patients had the test performed twice. All SACSTs were abnormal, defined as relative hepatic venous insulin (rHVI) levels >2 [11]. The rHVI levels ranged from 2 to 17, and maximum hepatic venous insulin (mHVI) levels ranged from 4 to 62. Two patients underwent 2 separate SACSTs, each localizing to discordant regions, consistent with nesidioblastosis. Four cases demonstrated insulin hypersecretion in multiple vascular territories, supporting a diagnosis of nesidioblastosis, while the remaining 3 cases showed insulin hypersecretion in a single vascular territory, consistent with insulinoma vs nesidioblastosis. Table 2 provides a summary of the SACST results. A detailed breakdown of rHVI and mHVI values by case and vascular territory is provided in the Supplementary Materials [20]. All 7 patients underwent abdominal imaging with computed tomography (CT), magnetic resonance imaging, or endoscopic ultrasound (EUS) to exclude a pancreatic mass consistent with insulinoma, and 6 out of 7 patients underwent prolonged fasts (ranging from 18 to 72 hours) to evaluate for insulinoma, which were negative.

**Table 2.** Selective arterial calcium stimulation testing results

Case	rHVI	mHVI (μU/mL)	Artery showing response	Localization territory
1	17	17	PSA and DSA	Pancreatic body
2	2.4	15	GDA and PSA	Pancreatic head and body
	8	14	GDA and PSA	Pancreatic head and body
3	13	13	PSA	Pancreatic body
	12.4	62	PSA, GDA, PHA, DSA, SMA	Greatest pancreatic body, multiple territories with response
4	2	5	PSA	Pancreatic body
5	2.75	15	GDA and PSA	Pancreatic head and body
6	3.9	27	GDA, PSA, CH	Pancreatic head and body
7	4	4	PSA	Pancreatic body

Abbreviations: CH, common hepatic artery; DSA, distal splenic artery; GDA, gastroduodenal artery; mHVI, maximum hepatic venous insulin concentration; PHA, proper hepatic artery; PSA, proximal splenic artery; rHVI, relative-fold increase in hepatic venous insulin concentration; SMA, superior mesenteric artery.

### Pathology Results After Pancreatic Surgery

Pathology was reviewed by an experienced pathologist specializing in pancreatic endocrine disease. Five of the 7 patients underwent pancreatectomy, with varied pathology results (Table 3). Of these, 2 cases had histopathological findings consistent with nesidioblastosis. While there are no universally accepted diagnostic criteria for adult nesidioblastosis, the diagnosis was made based on recognized histologic features—including islet cell hypertrophy, lobulated or irregular islet architecture, pleomorphic islet cell nuclei, and the presence of ductuloinsular complexes—in the appropriate clinical context [9, 10]. Case 4 demonstrated an increased number of pancreatic islets in some areas, which were enlarged, lobulated, and irregular, with some islets located near pancreatic ducts. The pathologist noted that these histomorphologic findings, combined with the clinical history, were consistent with nesidioblastosis. Additionally, patchy low-grade pancreatic intraepithelial neoplasia (PanIN) was observed. Case 5 also showed hypertrophic islet cells with pleomorphic nuclei and ductuloinsular complexes, consistent with nesidioblastosis, with no evidence of dysplasia or malignancy. The remaining cases did not report histopathologic findings consistent with nesidioblastosis. Case 1 had multifocal low-grade PanIN without any mention of nesidioblastosis. Case 2 showed patchy mild chronic pancreatitis and low-grade PanIN but no evidence of nesidioblastosis. Case 3 demonstrated PanIN-2, dilated pancreatic ducts, and 22 lymph nodes negative for malignancy but no neuroendocrine tumor or mention of nesidioblastosis.

**Table 3.** Pathology findings after pancreatectomy

Case	Pancreatectomy type	Pathology findings	Mention of nesidioblastosis on pathology report
1	Distal	Multifocal PanIN, no endocrine tumor	No
2	Distal	Patchy chronic pancreatitis, low-grade PanIN	No
3	Distal	PanIN-2, dilated pancreatic duct (0.4 cm), no neuroendocrine tumor	No
4	Distal and subsequent total	Increased islet cells with some enlarged, lobulated, and irregular consistent with nesidioblastosis, low-grade PanIN	Yes
5	Total	Hypertrophic islet cells and pleomorphic nuclei and formation of some ductuloinsular complexes consistent with nesidioblastosis	Yes

Abbreviation: PanIN, pancreatic intraepithelial neoplasia.

### PBH Recurrence After Pancreatic Surgery

Originally, 4 patients underwent a distal pancreatectomy, and 1 patient underwent a total pancreatectomy. Among the 4 patients who underwent partial pancreatectomy, 3 initially experienced complete resolution of PBH. However, symptoms recurred in all 3 cases: after 1 year in case 1 and after 7 years in cases 2 and 3. Despite recurrence, the symptoms were consistently milder than at initial presentation. The fourth patient who underwent a distal pancreatectomy (case 4) experienced minimal improvement and continued to have persistent symptoms, leading to a total pancreatectomy for further symptom control. This patient and the patient from case 5, who underwent total pancreatectomy, achieved complete resolution of PBH but subsequently developed insulin-dependent diabetes. Table 4 summarizes patient outcomes after pancreatic surgery.

**Table 4.** Patient outcomes after pancreatectomy

Case	Pancreatectomy type	Resolution of PBH symptoms after surgery	Time to recurrence of PBH after pancreatectomy (Years)	Current status
1	Distal	Yes	1	Mild PBH symptoms returned
2	Distal	Yes	7	Mild PBH symptoms returned
3	Distal	Yes	7	Mild PBH symptoms returned
4	Distal followed by total pancreatectomy	Improved but never resolved after distal pancreatectomy	NA	Insulin-dependent diabetes
5	Total	Yes	NA	Insulin-dependent diabetes

Abbreviations: NA, not applicable; PHA, proper hepatic artery.

Of note, 2 patients completed calcium stimulation tests but did not pursue pancreatic surgery (cases 6 and 7). Case 6 opted for RYGB reversal, which did not lead to improvement in symptoms; she continues on dietary and medical therapy. Case 7 chose to continue dietary and medical therapy for persistent PBH symptoms and has not pursued surgical intervention.

## Discussion

### Literature Review of Nesidioblastosis Associated With PBH

In 2005, Service et al were the first to report pathological findings suggestive of nesidioblastosis in patients with PBH [1]. In their study, 5 out of 6 patients with severe hypoglycemia post-RYGB were found to have β-cell hypertrophy and increased islet cell mass, leading the authors to propose nesidioblastosis as a significant contributor to PBH. There have been several case reports since then with findings suggesting nesidioblastosis. Patti et al also supported the role of nesidioblastosis by reporting on 3 patients who underwent partial pancreatectomy for PBH where 2 out of the 3 patients had a positive SACST [7]. Pathological examination revealed β-cell expansion in these patients. They hypothesized that altered gastrointestinal anatomy post-RYGB may lead to increased GLP-1 secretion, causing islet cell expansion, as this had been demonstrated in rodents [21]. Kim et al also reported a case of PBH after RYGB with a SACST consistent with nesidioblastosis, where the patient's hypoglycemia resolved following subtotal pancreatectomy. The histological examination confirmed diffuse β-cell hypertrophy [22].

Of note, Meier et al critically reevaluated the pancreatic specimens from the study by Service et al and found no significant increase in β-cell mass compared to controls [23]. Their findings argued against the consistency of nesidioblastosis as a pathological hallmark of PBH, suggesting that the condition might be overdiagnosed based on these histological criteria. Additionally, in a comment on Patti et al's case series mentioned earlier, Meier et al raised significant concerns regarding the interpretation of the histopathological findings presented [24]. Meier and colleagues pointed out that the criteria used to diagnose nesidioblastosis were not sufficiently stringent and that the observed β-cell changes might not necessarily indicate pathological hyperplasia. Instead, these changes could represent normal physiological variability, especially considering the lack of a standardized definition for nesidioblastosis in adults. However, Meier et al did not dispute the positive SACST results reported.

Nesidioblastosis is currently classified into 3 histologic forms: focal, diffuse, and atypical [25]. The focal form is characterized by clusters of abnormal islet cells confined to a specific region of the pancreas, while the diffuse form involves abnormal islets scattered throughout the entire pancreas. Cases that do not clearly align with either focal or diffuse patterns are categorized as atypical [25]. Histologic changes associated with nesidioblastosis can vary widely among patients. These may include large clusters of islet cells displacing the surrounding acinar tissue, the proliferation of islet cells originating from ducts (referred to as ductuloinsular complexes), and islet cell dysplasia or nesidiodysplasia. Additionally, scattered islet cells, predominantly β cells, may display enlarged, hypertrophic nuclei [25]. Although these histologic features can support a diagnosis of nesidioblastosis, none are pathognomonic. There are no universally accepted diagnostic criteria for pancreatic islet cell hyperplasia. Some researchers define it as an endocrine cell mass exceeding 2% of the total pancreatic mass in adults or 10% in infants [10]. The diagnosis often involves a degree of subjectivity. Therefore, none of the histologic features are pathognomonic for nesidioblastosis [9]. Accurate diagnosis requires a comprehensive evaluation of clinical, biochemical, and histopathological findings, highlighting the complexity of identifying this condition.

In our study of cases who underwent partial or complete pancreatectomy, only 2 out of the 5 patients had findings of nesidioblastosis reported in their pathology examinations despite all 5 patients exhibiting SACST results suggestive of nesidioblastosis. This discrepancy highlights the inherent challenges in making a definitive histologic diagnosis of nesidioblastosis. SACSTs offer a functional assessment that may detect clinically significant insulin dysregulation not always evident on pathology.

### Alternative Mechanisms of PBH and Surgical Outcomes

Davis et al conducted a study in which 6 patients with PBH underwent reversal of RYGB or conversion to gastric sleeve. All patients reported improvements in hypoglycemic episodes, and follow-up tests showed reduced postprandial glucose, insulin, and GLP-1 levels. The authors suggested that the pathophysiology of PBH is better explained by altered gastrointestinal anatomy and timing of carbohydrate absorption rather than nesidioblastosis [26]. The patients in the study did not undergo SACSTs.

A literature review by Xu et al compared surgical treatments for PBH, focusing on reversal of RYGB vs partial pancreatectomy. They found that RYGB reversal led to hypoglycemia resolution in 42/48 patients (88%). SACST data were not provided, but perhaps nesidioblastosis contributed to the failure of reversal to resolve hypoglycemia in the 12% of patients who had persistent hypoglycemia. In comparison, a different set of patients received partial pancreatectomy, which resolved hypoglycemia in only 27/50 (54%). Nesidioblastosis was reported on histopathology in 47/50 patients (94%) who underwent pancreatectomy, yet as discussed, the diagnosis can be subjective and lacks standardized criteria [15]. Notably, the higher rate of resolution of PBH with RYGB reversal indicates that nesidioblastosis is unlikely to be the primary mechanism of PBH.

### Results of SACSTs in PBH

A recent study by Ciudin et al explored the use of canagliflozin, a sodium-glucose cotransporter-2 inhibitor, as a treatment for PBH and completed SACSTs in 10 of the study patients to assess for nesidioblastosis [27]. All patients showed a normal insulin response to calcium stimulation with no significant increase in insulin levels in any arterial territory. This finding argues against nesidioblastosis as the primary cause of PBH in these patients as SACSTs typically show a diffuse pattern of increased insulin secretion in nesidioblastosis. Importantly, the study showed that patients with PBH can have normal SACST results.

The reason for the discordant results between the patients in the aforementioned study and our patients may be the severity of disease. In our case series, all 7 patients had a positive SACST. Notably, 2 patients (cases 2 and 3) underwent a repeat SACST that localized to different arterial territories, further supporting a diffuse pattern of β-cell hyperfunction. Our patients were selected for SACSTs as part of preoperative planning after exhausting dietary and pharmacologic options, whereas the patients in Ciudin's study were mainly analyzing the effects of sodium-glucose cotransporter-2 inhibitor and underwent a SACST as a secondary investigation [27]. It is possible that islet cell response to altered physiology and anatomy is a spectrum, with more severe cases developing nesidioblastosis over time.

Differentiating insulinoma from nesidioblastosis on a SACST alone can be difficult. A study by Thompson et al examined mHVI and relative-fold increase in rHVI obtained during a SACST as differentiators between insulinoma and nesidioblastosis [11]. mHVI refers to the highest absolute insulin concentration measured in the hepatic vein following selective calcium injection into a pancreatic artery, whereas rHVI measures the fold increase in hepatic insulin concentration over baseline. The study found that insulinomas compared to nesidioblastosis had a mean mHVI that was 22 times higher (778.6 vs 36.2, *P* = < .001) and an average rHVI that was 3.9 times higher (25.1 vs 6.4, *P* = < .001) [11]. However, there was a significant overlap between the 2 conditions. For instance, an rHVI >19 is 99% specific for insulinoma, yet an rHVI >2, though consistent with β-cell abnormality, is only 4.1% specific for insulinoma [11]. Baseline insulin variability can also distort rHVI, making it less reliable. This overlap highlights the need to consider mHVI alongside rHVI for better diagnostic accuracy. A very high mHVI, particularly above the optimal cutoff value of >83 μIU/mL, which has 83% sensitivity and 93% specificity, may suggest insulinoma over nesidioblastosis, as it reflects a marked insulin hypersecretion that is more characteristic of insulin-producing tumors than the more gradual insulin overproduction seen in nesidioblastosis [11]. Interestingly, all of our patients with suspected nesidioblastosis had an mHVI <62 μIU/mL, further supporting its role as a potential diagnostic marker for distinguishing between these conditions [20].

Since normal β cells should not respond to calcium gluconate stimulation, an rHVI >2 indicates β-cell hyperfunction (either hyperinsulinism from nesidioblastosis or insulinoma) [11, 12]. To further differentiate the 2 entities, additional tests and imaging are used. A 72-hour fast has a sensitivity approaching 100% for insulinoma [28]. A 48-hour fast also has an excellent sensitivity of 95% [29]. Additionally, imaging can help differentiate insulinoma and nesidioblastosis. EUS shows a high sensitivity of 87% in detecting insulinoma [30]. The sensitivity of thin-section arterial phase CT for detection is 94%, and the sensitivity for combined biphasic thin-section helical CT and EUS has been reported to be 100% [31]. Patients with recurrent hyperinsulinemic hypoglycemia who have a positive SACST but negative results from a 48- or 72-hour fast and normal imaging studies most likely have nesidioblastosis, such as our 7 patients.

Lastly, over the past decade, it has become evident that partial pancreatectomy does not lead to resolution of PBH in the long term. Since partial pancreatectomy has fallen out of favor as a treatment option for PBH, SACST testing in patients with PBH to evaluate for nesidioblastosis is now infrequently performed. Thus, routine use of SACSTs in patients with PBH is not recommended as it is invasive and most often will not lead to changes in PBH management.

## Conclusion

PBH remains a challenging condition to manage due to its complex and multifactorial pathophysiology. While some prior studies have dismissed the involvement of nesidioblastosis in PBH entirely, nesidioblastosis may play a role, particularly in more severe cases. However, it is likely just 1 part of a broader spectrum of pathological changes. Nesidioblastosis is likely underrecognized due to the infrequent use of SACSTs in PBH since this procedure is invasive and does not often alter our management. In addition, this study highlights the difficulties associated with making a histological diagnosis of nesidioblastosis, which is often hampered by variability in findings.

The persistence or return of hypoglycemia in many patients despite pancreatectomy—an intervention aimed at reducing β-cell mass—suggests that other mechanisms, such as altered gastrointestinal anatomy leading to higher incretin levels, bile acid composition changes, and alterations in counterregulatory hormones, play a more significant role in PBH's overall pathogenesis [3-5]. In our case series, it is interesting that hypoglycemia initially resolved in 3 of the 4 patients following pancreatic surgery, and in 2 patients, this resolution lasted 7 years before hypoglycemia returned. It is possible that the recurrence of hypoglycemia reflects the redevelopment of nesidioblastosis in the context of persistent unaltered Roux-en-Y anatomy.

While this case series is not intended to draw definitive conclusions or establish causality, our goal is to highlight clinical patterns and raise questions that may inform future research and guide patient care. Overall, our case series aligns with previous studies showing that partial pancreatectomy is not a preferred treatment option for PBH as it does not lead to long-term resolution of hypoglycemia. Our study also highlights that histological diagnosis of nesidioblastosis is difficult, and SACSTs can be a helpful functional tool to indicate the presence of nesidioblastosis. It also underscores the importance of careful patient selection for bariatric surgery and consideration of identified risk factors prior to RYGB.

## Disclosures

D.D.: no financial disclosures; H.L.: received compensation from Vogenx for clinical research and from Amylyx for clinical research and advisory board participation; P.T.: received consulting fees from Boston Scientific and Cook Medical, serves on the advisory board for Pneumonix, and has received grant funding from the National Heart, Lung, and Blood Institute, National Cancer Institute, and the American Heart Association (unrelated to this work).

## Data Availability

Original data generated and analyzed during this study are included in this published article or in the data repositories listed in References.

## References

[1] Service GJ, Thompson GB, Service FJ, Andrews JC, Collazo-Clavell ML, Lloyd RV. Hyperinsulinemic hypoglycemia with nesidioblastosis after gastric-bypass surgery. N Engl J Med. 2005;353(3):249‐254. 16034010 10.1056/NEJMoa043690

[2] Lee CJ, Clark JM, Schweitzer M, et al Prevalence of and risk factors for hypoglycemic symptoms after gastric bypass and sleeve gastrectomy. Obesity (Silver Spring). 2015;23(5):1079‐1084. 25866150 10.1002/oby.21042 PMC4414701

[3] Salehi M, Woods SC, D'Alessio DA. Gastric bypass alters both glucose-dependent and glucose-independent regulation of islet hormone secretion. Obesity (Silver Spring). 2015;23(10):2046‐2052. 26316298 10.1002/oby.21186 PMC4586360

[4] Laferrere B, Heshka S, Wang K, et al Incretin levels and effect are markedly enhanced 1 month after roux-en-Y gastric bypass surgery in obese patients with type 2 diabetes. Diabetes Care. 2007;30(7):1709‐1716. 17416796 10.2337/dc06-1549 PMC2743330

[5] Korner J, Bessler M, Inabnet W, Taveras C, Holst JJ. Exaggerated glucagon-like peptide-1 and blunted glucose-dependent insulinotropic peptide secretion are associated with roux-en-Y gastric bypass but not adjustable gastric banding. Surg Obes Relat Dis. 2007;3(6):597‐601. 17936091 10.1016/j.soard.2007.08.004 PMC2134840

[6] Lee CJ, Brown TT, Schweitzer M, Magnuson T, Clark JM. The incidence and risk factors associated with developing symptoms of hypoglycemia after bariatric surgery. Surg Obes Relat Dis. 2018;14(6):797‐802. 29678347 10.1016/j.soard.2018.01.028

[7] Patti ME, McMahon G, Mun EC, et al Severe hypoglycaemia post-gastric bypass requiring partial pancreatectomy: evidence for inappropriate insulin secretion and pancreatic islet hyperplasia. Diabetologia. 2005;48(11):2236‐2240. 16195867 10.1007/s00125-005-1933-x

[8] Rabiee A, Magruder JT, Salas-Carrillo R, et al Hyperinsulinemic hypoglycemia after roux-en-Y gastric bypass: unraveling the role of gut hormonal and pancreatic endocrine dysfunction. J Surg Res. 2011;167(2):199‐205. 21414635 10.1016/j.jss.2010.09.047 PMC3148142

[9] Sempoux C, Kloppel G. Pathological features in non-neoplastic congenital and adult hyperinsulinism: from nesidioblastosis to current terminology and understanding. Endocr Relat Cancer. 2023;30(9). 10.1530/ERC-23-0034 37279235

[10] Rindi G, Solcia E. Endocrine hyperplasia and dysplasia in the pathogenesis of gastrointestinal and pancreatic endocrine tumors. Gastroenterol Clin North Am. 2007;36(4):851‐865, vi. 17996794 10.1016/j.gtc.2007.08.006

[11] Thompson SM, Vella A, Thompson GB, et al Selective arterial calcium stimulation with hepatic venous sampling differentiates insulinoma from nesidioblastosis. J Clin Endocrinol Metab. 2015;100(11):4189‐4197. 26312578 10.1210/jc.2015-2404 PMC4702445

[12] Kaplan EL, Rubenstein AH, Evans R, Lee CH, Klementschitsch P. Calcium infusion: a new provocative test for insulinomas. Ann Surg. 1979;190(4):501‐507. 226013 10.1097/00000658-197910000-00009 PMC1344516

[13] Szczepanski JM, Hissong E, Manthei DM. Use of selective arterial calcium stimulation testing in identification of insulinoma in a patient after bariatric surgery. Lab Med. 2022;53(2):e40‐e43. 34480182 10.1093/labmed/lmab071

[14] Thompson GB, Service FJ, Andrews JC, et al Noninsulinoma pancreatogenous hypoglycemia syndrome: an update in 10 surgically treated patients. Surgery. 2000;128(6):937‐944, discussion 944-5. 11114627 10.1067/msy.2000.110243

[15] Xu Q, Zou X, You L, et al Surgical treatment for postprandial hypoglycemia after roux-en-Y gastric bypass: a literature review. Obes Surg. 2021;31(4):1801‐1809. 33523415 10.1007/s11695-021-05251-x

[16] Vanderveen KA, Grant CS, Thompson GB, et al Outcomes and quality of life after partial pancreatectomy for noninsulinoma pancreatogenous hypoglycemia from diffuse islet cell disease. Surgery. 2010;148(6):1237‐1245, discussion 1245-6. 21134557 10.1016/j.surg.2010.09.027 PMC3954513

[17] Sandoval DA, Patti ME. Glucose metabolism after bariatric surgery: implications for T2DM remission and hypoglycaemia. Nat Rev Endocrinol. 2023;19(3):164‐176. 36289368 10.1038/s41574-022-00757-5 PMC10805109

[18] Emous M, van den Broek M, Wijma RB, et al Prevalence of hypoglycaemia in a random population after roux-en-Y gastric bypass after a meal test. Endocr Connect. 2019;8(7):969‐978. 31234142 10.1530/EC-19-0268 PMC6612232

[19] Salehi M, Vella A, McLaughlin T, Patti ME. Hypoglycemia after gastric bypass surgery: current concepts and controversies. J Clin Endocrinol Metab. 2018;103(8):2815‐2826. 30101281 10.1210/jc.2018-00528 PMC6692713

[20] Dawes D, Trevedi P, Lawler H. Supplemental Data for: Nesidioblastosis in Patients with Severe Post-Bariatric Hypoglycemia: A Monocentric Case Series [Data set]. Zenodo. 2025. 10.5281/zenodo.16640221. PMC12342943 40799776

[21] Drucker DJ . Enhancing incretin action for the treatment of type 2 diabetes. Diabetes Care. 2003;26(10):2929‐2940. 14514604 10.2337/diacare.26.10.2929

[22] Kim K, Greenspan JL, Mehrara S, Wynne D, Ennis E. Nesidioblastosis: an uncommon complication seen post roux-en-Y gastric bypass. Endocrinol Diabetes Metab Case Rep. 2022:2022:22-0361. 10.1530/EDM-22-0361 PMC9875027 36571473

[23] Meier JJ, Butler AE, Galasso R, Butler PC. Hyperinsulinemic hypoglycemia after gastric bypass surgery is not accompanied by islet hyperplasia or increased beta-cell turnover. Diabetes Care. 2006;29(7):1554‐1559. 16801578 10.2337/dc06-0392

[24] Meier JJ, Nauck MA, Butler PC. Comment to: patti ME, McMahon G, Mun EC et al. (2005) severe hypoglycaemia post-gastric bypass requiring partial pancreatectomy: evidence for inappropriate insulin secretion and pancreatic islet hyperplasia. Diabetologia 48:2236-2240. Diabetologia. 2006;49(3):607‐608, author reply 609-10. 16450091 10.1007/s00125-005-0114-2

[25] Goossens A, Gepts W, Saudubray JM, et al Diffuse and focal nesidioblastosis. A clinicopathological study of 24 patients with persistent neonatal hyperinsulinemic hypoglycemia. Am J Surg Pathol. 1989;13(9):766‐775. 2669541

[26] Davis DB, Khoraki J, Ziemelis M, Sirinvaravong S, Han JY, Campos GM. Roux en Y gastric bypass hypoglycemia resolves with gastric feeding or reversal: confirming a non-pancreatic etiology. Mol Metab. 2018;9:15‐27. 29449181 10.1016/j.molmet.2017.12.011 PMC5869737

[27] Ciudin A, Sanchez M, Hernandez I, et al Canagliflozin: a new therapeutic option in patients that present postprandial hyperinsulinemic hypoglycemia after roux-en-Y gastric bypass: a pilot study. Obes Facts. 2021;14(3):291‐297. 33965935 10.1159/000515598 PMC8255644

[28] Service FJ, Natt N. The prolonged fast. J Clin Endocrinol Metab. 2000;85(11):3973‐3974. 11095416 10.1210/jcem.85.11.6934

[29] Hirshberg B, Livi A, Bartlett DL, et al Forty-eight-hour fast: the diagnostic test for insulinoma. J Clin Endocrinol Metab. 2000;85(9):3222‐3226. 10999812 10.1210/jcem.85.9.6807

[30] Puli SR, Kalva N, Bechtold ML, et al Diagnostic accuracy of endoscopic ultrasound in pancreatic neuroendocrine tumors: a systematic review and meta analysis. World J Gastroenterol. 2013;19(23):3678‐3684. 23801872 10.3748/wjg.v19.i23.3678 PMC3691045

[31] Gouya H, Vignaux O, Augui J, et al CT, endoscopic sonography, and a combined protocol for preoperative evaluation of pancreatic insulinomas. AJR Am J Roentgenol. 2003;181(4):987‐992. 14500214 10.2214/ajr.181.4.1810987

